# Molecular docking analysis of pyrrole derivatives with different breast cancer targets

**DOI:** 10.6026/9732063002001890

**Published:** 2024-12-31

**Authors:** Stephen Ilango, Girija K., Vasantha Kumar Kulothungan

**Affiliations:** 1 Department of Pharmaceutical Chemistry, Mother Theresea Post Graduate and Research Institute of Health Sciences (Government of Puducherry Institution), Gorimedu, Puducherry - 605006, India; 2 Department of Bioinformatics, Pondicherry University (Central University), Chinna kalapet, Puducherry 605014, India

**Keywords:** Molecular docking, molecular dynamics, sr9009, breast cancer target

## Abstract

Breast cancer is major risk of death in women. Hence, it is interest to document the molecular docking analysis of SR9009 (a pyrrole derivatives) with different breast cancer target protein targets such as HER2, Erα, PR, PI3K, AKT, Reverbα, BRMS1, Aromatase and mTOR, CDK4, CDK6, TK and Top II. Among 13 proteins, HER2, Erα, Aromatase, Reverbα, BRMS1 and Top II have good binding score affinity. Molecular Dynamic results show that significant higher binding energy for Reverb alpha + SR9009 complex found to be -220.618 +/- 19.145 kJ/mol compared to Reverb alpha + Doxorubicin complex found to be -154.812 +/- 18.235 kJ/mol. Molecular docking and dynamics analysis show that SR9009 is a potential drug candidate targeting Reverb alpha for anti-breast cancer activity.

## Background:

Breast Cancer (BC) occurs in every country of the world in women at any age after puberty and increasing rates in later life. According to World Health Organization (WHO), in 2022 there were 2.3 million women diagnosed with breast cancer and 670000 deaths globally. International Agency for Research on Cancer (IARC) as part of WHO reported that eight epidemiologic studies association between to shift work and breast cancer. Disruption of circadian rhythms can therefore associate with abnormal cell division occur in cancer. Influence of altered circadian rhythm on breast cancer was first noted in 1960s [1]. Environmental factor altered light and dark cycles such as those experienced by night shift workers can also affect incidence of BC. Hormonal receptor status also plays an important role in BC associated with night work higher expression of Positive estrogen receptor and positive Human Epidermal growth factor 2 receptor cancer [2]. The effect of exposure to light at night showed that there was a 14% increased risk of breast cancer in the highest light at night compared with lowest light at night [3].

REV-ERB alpha is core component of circadian clock and also significantly inhibited colony formation, cell cycle, cell migration and apoptosis in prostate cancer (PCa) cells through FOXM1 pathway blockade [4]. SR9009 has antitumor activity in small cell lung cancer by targeting Reverb alpha through the suppression of autophagy gene Atg5 [5]. Based on literature review, we have been taken Reverbα as a breast cancer target for docking analysis. Vinblastine a natural Vinca alkaloid that was initially identified from *Catharanthus roseus*. Used to treats breast cancer, Kaposi sarcoma, renal cell carcinoma and testicular cancer [6]. Doxorubicin is an anthracycline drug first extracted from Streptomyces paucities var. caesisus in the 1970 and used in the treatment of several cancers including breast, lung, gastric, ovarian, thyroid, non-Hodgkin's, Hodgkin's lymphoma, multiple myeloma, sarcoma and pediatric cancers [7]. Tamoxifen Citrate is used for the treats breast cancer for its selective estrogen receptor modulator action [8]. Targeted breast cancer protein like Human epidermal growth factor receptor 2 (HER2), Estrogen receptor alpha (ERα), Progesterone receptor (PR), Phosphatidylinositol-4,5-bisphosphate 3-kinase (PI3K), Threonine-protein kinase 1 (AKT), REV-ERB alpha (NR1D1), Breast cancer metastasis suppressor 1 (BRMS1), aromatase, Mammalian or Mechanistic target of rapamycin (mTOR), Cyclin-dependent kinase 4,6 (CDK4/6), Tyrosine-protein kinase and Topoisomerase II (TopII) [9]. HER2 is a membrane tyrosine kinase, oncogene that overexpressed and gene amplified in about 20% breast cancer and major driver for tumor development [10]. Abnormal estrogen receptor (ER) signalling can result in multiple disorders, including various cancers [11]. The activation of ERα results in increased expression of the PI3K/AKT/NF-κB signaling pathway, leading to tumor invasion and metastasis in breast cancer [12]. Larger exposure to progesterone hormone increases risk of breast cancer [13]. PI3K is family of lipid kinases and has been found to play a key regulatory role in many cellular processes including cell survival, proliferation and differentiation [14]. AKT1 increases cell proliferation through cell cycle protein like p21, p27, cyclin D1 and impairs apoptosis via p53 [15].

REV-ERBα is unique member of the nuclear receptor subfamily 1 group D member 1 (NR1D1) of proteins it has repressive function in cell proliferation and metabolism, which may be relevant during cancer pathogenesis [16]. BRMS1 metastasis suppressors may represent novel therapeutic targets for metastasis [17, 18]. Aromatase is the enzyme that catalyzes the conversion of androgens to estrogens, where estrogens are known important in the growth of breast cancer in both pre- and postmenopausal women [19]. Research has usually shown that activated mTOR signaling leads to an increase in tumor progression [20]. CDK4/6 is serine/threonine kinases that contain a 300-amino acid catalytic domain generally inactive. Numerous preclinical studies have revealed that cyclin D1-CDK4/6 essential factor in behind the tumorigenic potential of breast cancer cells [21]. Human Epidermal growth factor receptor is a classic Tyrosine-protein kinase, overexpressed in breast cancer tissues and associated with higher aggressiveness and poor clinical outcomes [22, 23] Topoisomerase II alpha which is a 170kd protein located at chromosome 17 is up-regulated by the proliferating cells and TOP 2 A potential roles as a target for anticancer drugs and prognostic marker in breast cancer [24]. Therefore, it is of interest to report research works on the anticancer activity of SR9009 has been reported but *in-silico* docking approach not have been reported in breast cancer targets and attempt has been made to evaluate the clear mechanism of action SR9009 with breast cancer targets through *in-silico* approach.

## Methodology:

Molecular docking aims to predict the ligand-receptor complex through computer-based methods [25]. Molecular Docking has become an essential aspect of *in-silico* drug development in recent years. Pre-docking steps ligand preparation, protein preparation and homology modeling.

## Hardware, software & Website:

Hardware been used in laptop with Intel® CoreTM i7-1255U RAM 16.0 GB @ 1.70 GHz, 64-bit operating system at Windows 11.

Software for molecular docking analysis using MGL tools 1.5.7 were downloaded from (https://ccsb.scripps.edu/mgltools/downloads/ ) [26]. To remove water molecule, particular chain of protein and converted into pdb format using Pymol 2.5.4 were downloaded from (https://pymol.org/edu/ ) [27]. To convert the 2D structure into 3D structure using Avogardo 1.2.0 version software were downloaded from (https://avogadro.cc/ ) [28]. To find the missing sequence of proteins were filled using Modeller 10.5 software was downloaded from (https://salilab.org/modeller/download_installation.html) [29]. Interaction between proteins and ligands visualized using Chimera X were downloaded from (https://www.cgl.ucsf.edu/chimerax/download.html) [30]. For molecular dynamics, Groningen machine for chemical simulation (Gromacs) software version 2020.4 has been used. ADME properties were calculated using Swiss ADME (http://www.swissadme.ch/) [31]. In order to take chemical structure of ligands were downloaded from Pubchem database (https://pubchem.ncbi.nlm.nih.gov/) [32]. Protein structure was downloaded from RCSB protein Data Bank (PDB) (http://www.rcsb.org/pdb/) [33], Active site of proteins were selected from PDBsum (https://www.ebi.ac.uk/thornton-srv/databases/pdbsum/) [34] and computed atlas of surface topography of proteins (CASTp) (http://sts.bioe.uic.edu/castp/index.html?201l) [35]. For Toxicity assessment, OSRIS property explorer open-source program was downloaded from (https://www.organic-chemistry.org/prog/peo/) [36]. Missing loop of proteins sequences were filled using emboss needle (https://www.ebi.ac.uk/jdispatcher/psa/emboss_needle) [37]. Energy minimization of proteins was done using Yasara online webtool (https://www.yasara.org/minimizationserver.htm) [38] and proteins verification done using Ramachandran plot analysis in PDBsum website link which has been mentioned above. Regarding Molecular dynamics study Ligand topology performed using Automated Topology Builder (ATB) web server (https://atb.uq.edu.au/) [39].

## Screening of ADME, physiochemical properties, drug likeness prediction and toxicity assessment:

ADME screening, Physiochemical properties and drug-likeness evaluation were done using free website using Swiss-ADME, which has been developed by Swiss Institute of Bioinformatics. Drug likeness properties were determined by Lipinski [40], Ghose [41], Veber [42], Egan [43] and Muegge [44] rules of 5 screening. Abbot bioavailability score was be determining the bioavailability of ligands. OSRIS property explorer were used to determine toxicity profile of ligands for mutagenic, tumorigenic, irritant and reproductive effective by comparing the colour code, determine which has been toxicity will be in Red or safest in green colour.

## Ligand preparation:

The structure of ligands and structural information was obtained from PubChem database. SR9009 (PubChem CID: 57394020), Vinblastine (PubChem CID: 13342), Doxorubicin (PubChem CID: 31703) and Tamoxifen citrate (PubChem CID: 2733525) were chosen as a ligand. SR9009 taken as test and Vinblastine, Doxorubicin and Tamoxifen citrate were chosen as standard. 2D structure of ligand (Tamoxifen Citrate and Vinblastine] converted into 3D structure using Avogadro software. After conformation with Lipinski rule of 5 and Toxicity study result of test ligand (SR9009) proceed to perform the docking analysis [45].

## Macromolecule preparation:

In the Present study, different breast cancer target proteins were retrieved from RCSB PDB database. Target protein having X-Ray diffraction resolution size not more than of 3.0 Å were used [46]. Further, water molecules and hetero groups were removed from protease structure using Pymol.

## Homology modelling:

Protein having missing residues were constructed by homology modelling using MODELLER (Version -10.5) and sequence of proteins were considered as a template were obtained from *fasta* sequence in text format from RCSB PDB website. Missing sequence of the protein were detected by Pymol and protein sequence gap aligning them using EMBOSS needle. Later that energy minimization was done by using Yasara online web tool. Modelled structure of protein was validated using PROCHECK [47] to check stereo chemical quality of protein based on Ramachandran Plot. If proteins have more than 90 percent and G-Factors has more than -0.5 these results suggest good structure quality of protein ready for molecular modelling [48].

## Molecular docking:

Autodock tools 1.5.7 version software was used for docking analysis. Active site for proteins was predicted using CASTp server and PDBsum common active site were selected [49]. Polar hydrogen atom was added to the protein targets and Kollman united atomic charges were added. The pdbqt charge file of protein and ligand are prepared [50]. The targets grid map calculated and set to 60 x 60 x 60 points with grid spacing of 0.375 Å. Grid parameter file (Gpf) and Docking parameter file (dpf) file were created to run auto grid and auto dock application. The Genetic Algorithm (GA), 25 runs will be made to get the desired docking conformation. Lowest binding docking score with hydrogen bonding formation normally taken as a best docking score and visualized using Chimera X [51].

## Molecular dynamics simulation study:

Molecular Dynamics (MD) simulation was conducted using pdb2gmx module of GROMACS 2020.2 version. Ligand topology was selected from ATB server added heavy atoms. Prepared system was first vacuum minimized for 1500 steps using the steepest descent algorithm. Structure was solvated in a cubic periodic box with a water simple point charge (SPCE) water model [52]. By using various parameters provided by GROMACS 2020.4 software package including the protein root mean square deviation (RMSD), root mean square fluctuations (RMSF), radius of gyration (RG), solvent accessible surface area (SASA), Hydrogen bonding (H-Bond), Principal component analysis (PCA), Free energy landscape (FEL) and Molecular Mechanics Poisson-Boltzmann surface area (MM-PBSA) approach was employed to understand binding free energy of an affinity with targeted protein over 100ns simulation time. A GROMACS utility g_mmpbsa was employed to estimate the binding free energy [53].

## Results and Discussion:

Values of ADME properties of ligands shown in (Table 1) indicate gastrointestinal absorption shows high only in SR9009 and low in vinblastine, doxorubicin and Tamoxifen citrate. No BBB per meant was observed for all ligands. Vinblastine, doxorubicin was shown Pgp substrate. For SR9009 most of cytochrome P450 isoenzyme was inhibited only CYP1A2 isoenzyme was not inhibited. Vinblastine inhibited only CYP3A4 isoenzyme. Doxorubicin shows no inhibition of cytochrome P450 enzymes and Tamoxifen citrate inhibited only CYP2D6 isoenzyme. Values of Physiochemical properties and lipophilicity of ligands were shown in (Table 2) indicate that molecular weight of test ligand SR9009 shows 437.94 which is fewer than 500, good ligand for docking analysis as per Lipinski rule of five. For vinblastine, doxorubicin and Tamoxifen citrate were shows 811, 543.52 and 563.64 which is more than 500 even through have been taken as standard ligands for docking analysis. Water solubility SR9009 shows moderately soluble, vinblastine shows poorly soluble, doxorubicin shows soluble and tamoxifen citrate shows moderately soluble. Lipophilicity shows SR9009 (3.45), vinblastine (3.79), doxorubicin (0.52) and tamoxifen citrate (4.11). Toxicity profile of ligands red colour shows in Doxorubicin was found to be irritant and Tamoxifen citrate was found that reproductive toxicities, green colour shows to SR9009 and Vinblastine was found to no toxicological features were obtained from OSRIS predictions. Overall, test ligand SR9009 only obeys Lipinski rule of five no violation but Vinblastine, Doxorubicin and Tamoxifen citrate has shown violation. Even though we have taken Vinblastine, Doxorubicin and Tamoxifen citrate as standard ligands for molecular modelling studies owing to it have been available as standard treatment drugs in breast cancer patients. Regarding Molecular docking analysis active site prediction has done by 2 servers Castp and PDBsum the common active were selected shown in (Table 3).

Breast cancer targets HER2, ERα, PR, PI3K, AKT, REV-ERBα, BRMS1 and mTOR, Aromatase, CDK4, CDK6, TK and TopII. Among 13 proteins, SR9009 has showed higher binding affinity against 6 proteins such as HER2, ERα, REV-ERBα, BRMS1, Aromatase and TopII were shown in (Table 4), compared to vinblastine, doxorubicin and Tamoxifen citrate. The protein which are docked with SR9009 are HER2 (Figure 1A) interact with Hydrogen bond residue Phe 731 and Lys 753, Erα (Figure 1B) interact with Lys 520, REV-ERBα (Figure 1C) interact with Lys 473, BRMS1 (Figure 1D) interact with Arg 57, Arg 82 and Glu 85, Aromatase (Figure 1E) interact with Arg 435, Arg 145, Arg 115 and Trp141, TopII (Figure 1F) interact with Gln 301 and Ile 302 were found to form hydrogen bond with these targets and showed best higher docking score as good binding energy of -7.4kcal/mole, -7.65kcal/mole, -7.7kcal/mole, -7.64kcal/mole, -10.6kcal/mole and -6.78kcal/mole compared to Vinblastine, Doxorubicin and Tamoxifen citrate. As expected, confirmed that SR9009 (Test) have good binding affinity in breast cancer targets compared to doxorubicin, vinblastine and tamoxifen citrate through docking analysis.

Docking analysis confirm that ligand SR9009 have good binding affinity compared to vinblastine, Doxorubicin and Tamoxifen citrate and it will expect to have good activity *in-vitro* and *in-vivo* for antiproliferative activity with these 6 breast cancer targets. We hypothesis that as per the molecular docking results 6 targeted proteins has good binding score, Reverbα among one of them has been chosen for MD simulation studies for circadian targeted pathway in breast cancer activity through molecular simulation approach and also have good binding (-7.7 kcal/mole) affinity in docking analysis. Among 3 standards Doxorubicin, Vinblastine and Tamoxifen citrate, Doxorubicin has been chosen for MD simulation based on best docking score among standards. 100ns MD simulation research was used to assess the stability of the docked Reverb alpha (PDB ID: 3N00) and SR9009 complex. To obtain an accurate result, we computed SR9009 and Doxorubicin for last 50 ns with dt 1000 frames. For MD simulation results we used 3 complex systems APO (Reverbα only) in grey colour as control, Drug (SR9009 + Reverbα) in blue colour as Test and Standard (Doxorubicin + Reverbα) in red colour reveals.

## Root mean square deviation (RMSD):

Result indicates that all 3 complex system, Reverbα, SR9009 + Reverbα and Doxorubicin + Reverbα reached equilibrium within 10ns. At 10ns, all 3 system rise equilibrium and after that remain stable after simulation time. After 100ns simulation, average RMSD values reveal that Reverbα only is 0.63 ± 0.10nm, SR9009 + Reverbα is 0.65 ± 0.10nm and Doxorubicin + Reverbα is 0.62 ± 0.08. For all 3 complex system has similar lower value indicate that stable during 100ns simulation period. SR9009 + Reverbα (Blue colour) complex attain maximum at 0.83nm at 94ns and further decreases at 0.65nm at 97ns remain stable were shown in Figure 2 (see PDF) (RMSD) respectively.

## Root Mean square fluctuation (RMSF):

Average values of RMSF for Reverb alpha only (0.22 ± 0.13 nm), Reverb alpha + SR9009 complex (0.30±0.23 nm) and Reverb alpha + Doxorubicin complex (0.18 ± 0.12 nm) was observed over 100ns simulation period. Highest fluctuation at 1.2nm at 40 to 50 residue after that remain stable and slight fluctuation at 0.7nm at 100 to 150 residue for SR9009 + Reverbα complex shown in blue colour after that no further fluctuation has been occurred remain stable were shown in Figure 3(see PDF) (RMSF). RMSF shows lower value, loosely ordered and low fluctuations indicate the more stability but SR9009 + Reverbα in region of 40 - 50 residue shows high fluctuation indicate least stable. Highest fluctuation indicates the loosely ordered sheet and helices Remaining residues shows least fluctuations indicate that more stable.

## Radius of gyration (Rg):

Results indicated that dynamic stability and compactness of 3 complex system shown in Figure 4 (see PDF) (Rg). Average values of Reverb alpha only (1.96 ± 0.05 nm), Reverb alpha + SR9009 complex (2.13 ± 0.03 nm) and Reverb alpha + Doxorubicin complex (1.97 ± 0.02 nm) over 100ns simulation. Values indicate that Reverb alpha only shows (1.96 ± 0.05 nm) lower value reveals more compactness by comparing Reverb alpha + SR9009 and Reverb alpha + Doxorubicin. Overall Rg values were maintained at 2 to 2.2 nm fluctuation were maintained indicated that stability over simulation period.

## Solvent accessible surface area (SASA):

As part of the surface of protein that can interact with solvent molecule. Average results indicate for 3 complex system shows that Reverb alpha only (137.07 ± 5.91 nm), Reverb alpha + SR9009 complex (160.45 ± 4.77 nm) and Reverb alpha + Doxorubicin (137.66 ± 4.23 nm). Reverb alpha, Reverb alpha + Doxorubicin system reveals that surface area exposure reduced. While Reverb alpha + SR9009 complex system increases surface area of solvent accessibility. We have observed that surface areas in the range were maintained between 135 -165 nm2 for all complexes over simulation period were shown in Figure 5 (see PDF) (SASA).

## Hydrogen bond analysis:

To performed in Ligand-Target complex binding stability over 100ns and time dependent behaviour of intra and inter hydrogen bond for 3 complex system Reverb alpha, SR9009 and doxorubicin complex shown in Figure 6a (see PDF) (Intra HB) and Figure 6b (see PDF) [Inter HB]. Reveals that time-dependent behaviour of intra-hydrogen bonds values of Reverb alpha (187.92 ± 8.47 nm), Reverb alpha +SR9009 (173.32 ± 7.27 nm) and Reverb alpha + Doxorubicin (186.17 ± 7.41 nm). For all 3 system intra-hydrogen bond were formed. Inter-hydrogen bonds stabled during simulation and maintained by 1 to 5 hydrogen bonds for Reverb alpha + SR9009 complex and 1 to 7 hydrogen bonds for Reverb alpha + Doxorubicin complex. Overall, both intra and inter-hydrogen bond were formed in both SR9009 and doxorubicin complex formed hydrogen bond done essential role in the stabilization of protein-ligand interaction. Drug complex system may be potential drug against Reverbα.

## Principal component analysis (PCA):

Eigenvector (EV) play vital role in the global motion of protein molecule. To study the conformational dynamics of reverb alpha, SR9009 and doxorubicin during simulation shown in Figure 7 (see PDF) (PCA). Time evolutions of PCA Plot find that overall flexibility of the Reverb alpha alone, Reverb alpha + SR9009 and Reverb alpha + Doxorubicin complex. Plot predicts that lower number of movements observed in doxorubicin and did not significantly affect the target conformation and dynamics thus supporting stability of the complex.

## Free energy landscapes (FELs):

Here we generated FEL plots for PC1 and PC2 shown in Figure 8 (see PDF) (FELs) where deeper blue regions indicate a more stable protein conformation with lower energy. The plots indicate energy values ranging from 0 to 16 kJ/mol and 0 to 20 kJ/mol throughout the simulation of Reverb alpha + SR9009 and Reverb alpha + Doxorubicin complex respectively. The FEL plots reveal that the SR9009 and Doxorubicin complex display a single global minimum, confined to a large local basin. These findings predicted that SR9009 + Reverb alpha in (B) and Doxorubicin + Reverb alpha in (C) do not cause any significant conformational changes in the target structure indicate that more stability compared to Reverb alpha only (A).

## Molecular Mechanics/Poisson-boltzmann surface area (MM-PBSA):

GROMACS utility g_mmpbsa tool of determine the binding free energy. Calculate the total binding energy for both SR9009 + Reverbα complex shows -220.618 +/- 19.145 kJ/mol higher binding free energy compared to Reverb alpha + Doxorubicin complex shows -154.812 +/- 18.235 kJ/mol. Meanwhile electrostatic energy has been shows higher value -199.437 +/- 30.713 kJ/mol for SR9009 + Reverbα complex compared to Reverb alpha + Doxorubicin complex shows -34.038 +/- 10.298 kJ/mol has been shown in (Table 5). MD simulation results reveal that Reverbα + SR9009 complex indicate more stability over 100ns simulation time compared to Reverbα + doxorubicin complex. Overall, it indicates that SR9009 + Reverbα complex reveals stronger system stability has been found that SR9009 tightly bind to the Reverbα over 100ns simulation period compared to standard complex system. Finally results of docking analysis and MD simulation indicate that Reverbα + SR9009 complex more significant stable binding interaction compared to Reverbα + doxorubicin complex. SR9009 can be considering a clinical candidate molecule act on Reverbα target with high affinity based on results.

## Conclusion:

Molecular docking and simulation analysis show SR9009 has higher binding affinity with breast cancer protein target Reverb alpha for further consideration and validation.

## Figures and Tables

**Figure 1A:**
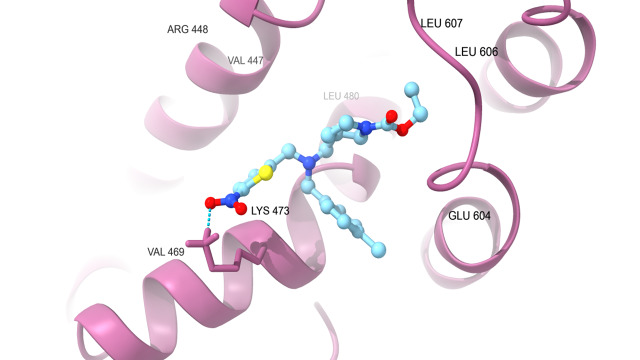
Docking analysis of HER2

**Figure 1B:**
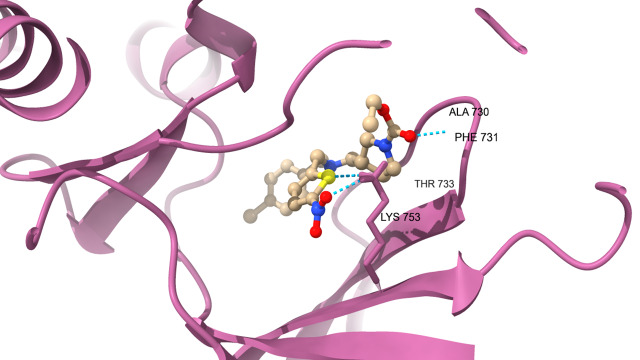
Docking analysis of ERalpha

**Figure 1C:**
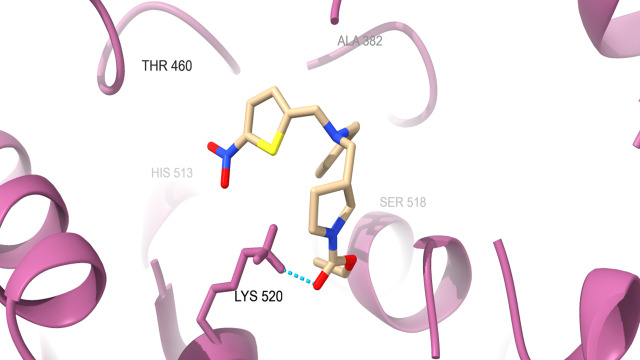
Docking analysis of Reverb alpha

**Figure 1D:**
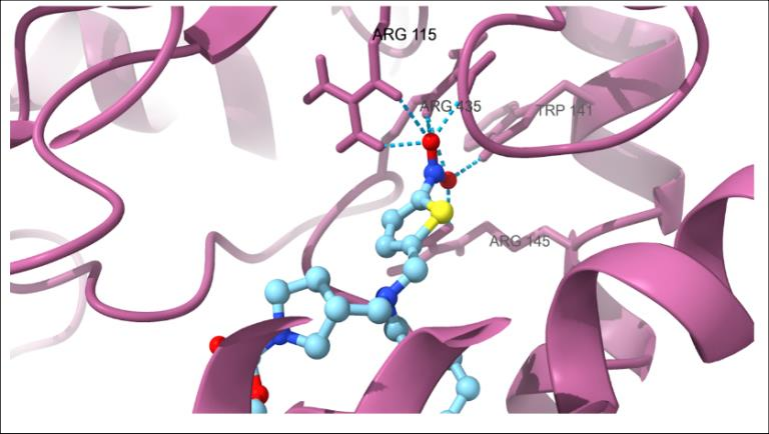
Docking analysis of BRMS1

**Figure 1E:**
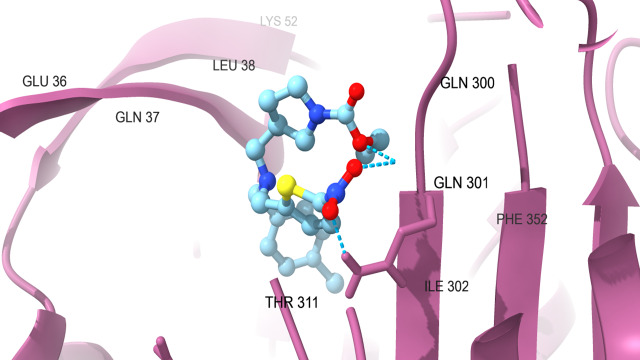
Docking analysis of aromatase

**Figure 1F:**
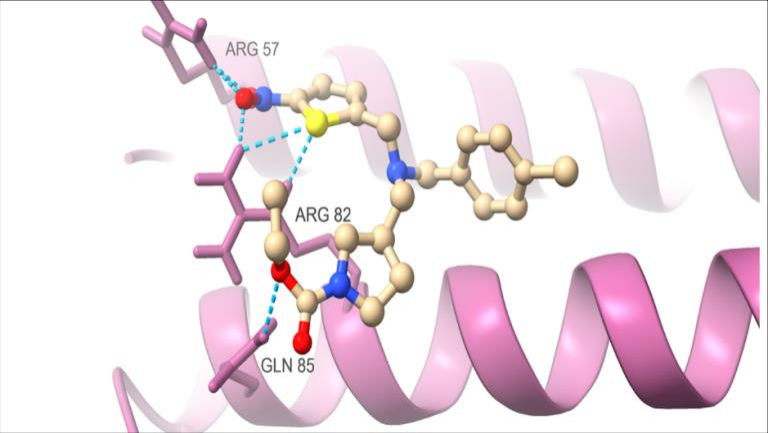
Docking analysis of TopII

**Table 1:** ADME & Drug likeness properties of Ligands

**Properties**	**Sr9009**	**Vinblastine**	**Doxorubicin**	**Tamoxifen citrate**
**ADME PROPERTIES**				
GI absorption	High	Low	Low	Low
BBB permeant	No	No	No	No
Pgp substrate	No	Yes	Yes	No
CYP1A2 inhibitor	No	No	No	No
CYP2C19 inhibitor	Yes	No	No	No
CYP2C9 inhibitor	Yes	No	No	No
CYP2D6 inhibitor	Yes	No	No	Yes
CYP3A4 inhibitor	Yes	Yes	No	No
Log Kp [cm/s]	-5.84	-8.49	-8.71	-7.71
**DRUG LIKENESS - LIPINSKI rule of 5**				
Lipinski violation	0	2	3	1
Ghose violation	0	3	2	3
Veber violation	0	1	1	2
Egan violation	0	1	1	1
Muegge violation	0	4	3	1
Bioavailability score	0.55	0.17	0.17	0.56

**Table 2:** Physiochemical properties & Lipophilicity of Ligands

**Properties**	**Sr9009**	**Vinblastine**	**Doxorubicin**	**Tamoxifen citrate**
**PYSIOCHEMICAL PROPERTIES**				
Molecular Formula	C20H24ClN3O4S	C46H58N4O9	C27H29NO11	C32H37NO8
Molecular weight	437.94	810.97	543.52	563.64
Hydrogen Bond Donor	2	3	6	4
Hydrogen Bond acceptor	5	11	12	9
Rotatable Bond	10	10	5	13
Molar Refractivity	120.13	232.52	132.66	157.19
Water Solubility Log S (ESOL]	Moderately soluble	Poorly soluble	Soluble	Moderately soluble
Topological Surface area (A)	106.84	154.1	206.07	144.6
**LIPOPHILICITY**				
TPSA	106.84	154.1	206.07	144.6
iLOGP	3.62	5.13	2.58	4.6
XLOGP3	4.41	3.88	1.27	2.85
WLOGP	4.11	2.85	-0.32	4.75
MLOGP	2.44	2.35	-2.1	2.75
SILICOS-IT Log P	2.66	4.72	1.17	5.99
Consensus Log P	3.45	3.79	0.52	4.11

**Table 3:** Common active site for protein determine by computed atlas of surface topography of proteins (Castp) and PDBsum

**S.no**	**Protein**	**Protein code**	**Active site residue**
1	HER2	7PCD	Leu726, Val734, Pro761, Ala775, Asp863, Gly732, Lys753, Glu770, Ser768
2	ERα	6V87	Leu428, Met343, Phe404, Gly521, Leu525, His524, Asp351, Ala350, Thr347
3	PR	1A28	800Thr, 886Gln, 889Leu, 890Tyr, 893Asn, 894Thr, 745Asp, 748Ile, 749Thr, 752Gln
4	PI3K	6NCT	Sel126, Lys208, Leu277, Phe200, Val109, Ale173, Asp124, Asp434(A)
5	AKT	3MV5	Val163, 179Lys, 195Thr, 227Met, 228Glu
6	REV-ERBα	3N00	Ala474, Phe477, Asp549, Ser551, Arg596, Asn599, Asn600, Ser603, Glu604
7	BRMS1	2XUS	Glu54[A], Arg55[A], Ser58[A], Glu59[A], Leu83[B], Leu86[B], Arg87[B], Arg89[B]
8	mTOR	4JT6	Val2240[B], Tyr2225[B], Asp2195[B]
9	Aromatase	3EQM	Met107, Arg115, Ile132, Ile133, Phe134, Trp141, Arg145, Trp224, Val370, Glu483
10	CDK4	2W96	Arg61, Asp99, Glu144, Ser166, Val176, Arg181, Tyr191
11	CDK6	1BI7	Gly22, Ala22, Tyr24, Ala162, Phe164, Arg186
12	TK	1QCF	Leu89, Tyr90, His96, Asn135, Phe150, Ala164, Ser247, Glu339
13	TopII	1PVG	Glu19, His20, Asp65, Lys147, Ala146, Gly145, Gln365, Arg141, Ser127, Tyr144

**Table 4:** Docking analysis of SR9009, doxorubicin, vinblastine & tamoxifen citrate with different breast cancer targeted proteins

	**Name of the protein**	**Protein**	**Ligand**	**Binding energy**	**Binding energy [vinblastine]**	**Binding energy [doxorubcin]**	**Binding energy [tamoxifen citrate]**
**S.NO**		**Code**	**Name**	**[SR9009]**			
1	HER2	7PCD		-7.4	-5.97	-7.17	-0.23
2	ERα	6V87	SR9009	-7.65	-6.75	-7.66	1.21
3	PR	1A28		-6.14	-6.74	-6.48	-1.7
4	PI3K	6NCT		-8.85	-8.87	-8.75	-2.41
5	AKT	3MV5	DOXORUBICIN	-6.03	-5.56	-8.05	0.53
6	REV-ERBα	3N00	[STD]	-7.7	-5.27	-7.4	-0.35
7	BRMS1	2XUS		-7.64	-3.75	-6.68	-3.55
8	mTOR	4JT6	VINBLASTINE	-7.21	-5.54	-7.79	-1.81
9	Aromatase	3EQM	[STD]	-10.6	-5.22	-8.9	-3.97
10	CDK4	2W96		-6.02	-8.05	-7.48	0.43
11	CDK6	1BI7		-7.92	-6.71	-8.4	-0.75
12	TK	1QCF	TAMOXIFEN CITRATE	-7.7	-9.41	-7.3	-0.8
13	TopII	IPVG $ 6ZY8	[STD]	-6.78	-6.6	-6.55	-3.69

**Table 5:** Molecular Mechanics/Poisson-Boltzmann surface binding energy for Reverbα + SR9009 complex and Reverbα + Doxorubicin complex

**DRG (Reverb alpha + SR9009)**	-199.437 +/- 30.713 kJ/mol	-220.618 +/- 19.145 kJ/mol
**STD (Reverb alpha + Doxorubicin)**	-34.038 +/- 10.298 kJ/mol	-154.812 +/- 18.235 kJ/mol
